# 한국 농촌 후기 청소년 여성의 자살 생각에 영향을 미치는 요인

**DOI:** 10.4069/kjwhn.2020.06.15

**Published:** 2020-09-29

**Authors:** Hae Kyung Jo, Hyun Kyoung Kim

**Affiliations:** 1 Department of Nursing, Jeonju University, Jeonju, Korea; 1 전주대학교 간호학과; 2 Department of Nursing, Kongju National University, Kongju, Korea; 2 국립공주대학교 간호학과

**Keywords:** Adolescent, Depression, Female, Suicide, Violence, 청소년, 우울, 여성, 자살, 폭력

## Abstract

**Purpose:**

This study aimed to examine factors affecting suicidal ideation among female late adolescents from a Korean rural province.

**Methods:**

A descriptive cross-sectional design involving self-reported questionnaires was used. The data were collected from female adolescents aged 18 to 22 years, from high schools, universities, churches, and local community centers. The sample consisted of 197 female late adolescents from three rural areas in Korea. The Center for Epidemiological Studies-Depression Scale and the Suicidal Ideation Scale were used to measure depression and suicidal ideation, respectively.

**Results:**

Suicidal ideation was correlated with having a specific suicide plan (r=.65, *p*<.001), depression (r=.39, *p*<.001), subjective well-being (r=–.46, *p*<.001), ever experienced violence (r=.15, *p*=.029), and age (r=-.21, *p*=.003). Factors affecting suicidal ideation among rural female adolescents were having a specific suicide plan (β=.56, *p*<.001), subjective well-being (β=–.23, *p*=.001), and suicide of a close person (β=.12, *p*=.035). These factors explained 48.0% of the variance in suicidal ideation (F=37.62, *p*<.001).

**Conclusion:**

This study demonstrated that affective factors and personal experiences regarding suicide were critical indicators for suicidal ideation among female late adolescents. These factors should be managed and tailored nursing should be offered to prevent suicide and enhance the psychiatric health of female late adolescents.

## Introduction

### 연구 필요성

우리나라 청소년의 자살률은 2018년 통계에서 인구 10만 명당 7.8명으로 세계적으로 가장 높은 수준이며, 2007년 이후 지속적으로 10세에서 24세 사이 연령대의 사망 원인 1위가 자살인 것으로 나타나 그 심각성을 확인할 수 있다[1]. 전 연령대에서 남성의 자살률이 높고 청소년기의 자살자 성비 또한 남성이 58.3%로 여성의 41.7%에 비해 높지만, 다른 연령대에 비해서는 여성의 비율이 상대적으로 높다[1]. 또한 우울과 스트레스를 가진 자살 잠재군은 오히려 여성 청소년이 20.2%로 남성 청소년의 13.4%에 비해 높은 수준을 보이고 있다[2].

자살은 단독의 사건적 개념이라기 보다는 자살 생각(suicide ideation), 자살 계획(suicide plan), 자살 시도(suicide attempt) 등, 자살에 이르는 일련의 과정이라는 관점으로 볼 수 있다[3]. 자살 생각이란 자신의 생명을 끊으려는 생각을 의미하며, 자살 계획은 자살을 하려는 의도, 자살 시도는 죽음에 이르지 않은 자살 행동을 의미한다[3]. 2019년 청소년 통계 조사에 의하면 남자 청소년의 자살 생각 비율이 11.0%인 것에 비해 여성 청소년의 자살 생각은 15.4%로 더 높았고, 자살 시도 비율도 여성 청소년이 2.6%로 남성 청소년의 2.3%에 비하여 높았다[4]. 따라서 자살 생각은 자살 시도와 자살로 이어질 수 있으므로 자살 생각에 영향을 미치는 요인을 파악할 필요가 있다.

청소년기는 이차 성징으로 인한 변화와 정체감의 확립에 젠더의 영향력이 증폭되는 시기이며, 생물학적으로나 사회문화적으로 민감하게 접근해야 하는 시기이다[4]. 여성 청소년 자살 생각의 영향요인을 조사하고 예방적 중재를 적용하기 위해서는 성 인지적 관점을 가지고 접근해야 할 필요가 있다[5]. 청소년은 다른 연령대에 비해 정신건강 문제보다 일시적인 정서 상태에 의한 자살 생각이나 현실을 도피하기 위한 수단으로서의 자살 생각이 많다[6]. 또한 대중매체의 영향을 많이 받아 유명인들을 모방하는 자살이 많으며, 자살의 방법으로 치명적인 방법을 선택하는 경우가 많다는 특징을 가지고 있다[6].

청소년기의 자살 생각에는 다양한 외재적, 내재적 영향요인이 작용한다고 밝혀져 왔다[7]. 청소년 자살 생각의 내재적 영향요인으로는 연령, 우울, 불안, 외로움, 낮은 안녕감, 무망감(hopelessness) 등이 있었으며[4,8], 외재적 영향요인으로는 사회적 지지, 폭력 경험, 주변인의 자살 경험, 경제적 환경 등이 나타났다[3,7,9,10]. 자살 생각의 내재적 영향요인 중 직접적 요인으로 지적되고 있는 우울은[3] 여성 청소년에게서 더 높게 나타나 심리적 어려움을 겪는 여성 청소년이 다수임을 알 수 있다[1]. 청소년의 정서 상태는 자신의 삶에 대한 전반적 평가인 주관적 안녕감을 사회적 지수로 흔히 나타내는데[11], 여성 청소년기의 삶에 대한 만족 정도인 안녕감을 자살 생각의 주요 영향요인으로 보고하고 있다[8]. 남성 청소년에 비해 여성 청소년은 충동적인 자살이 적어 구체적인 자살 계획이 자살 생각의 영향요인으로 작용한다[12]. 자살 생각의 외재적 영향요인 중 폭력 경험은 가정 폭력과 학교 폭력 모두가 영향을 미친다고 보고되고 있으며[12], 환경적 영향을 많이 받는 청소년의 특성상 가까운 주변인의 자살을 경험하는 것이 위험요인으로 작용한다[6]. 반면 여성 청소년은 소속감과 사회적 교류를 중요시하기 때문에 사회적 지지가 자살 생각에 보호요인으로 작용한다[12]. 그러므로 선행 연구에서 주로 여성 청소년에게 특징적으로 영향을 주었던 요인들을 통합하여 확인할 필요가 있다.

기존 연구들에 의하면 청소년 자살은 전기 청소년기(13-15세)나 중기 청소년기(16-18세)보다 후기 청소년기(19-22세)에[7] 더 많이 발생하였으며[10,13],전 세계적으로 10배 이상 높은 것으로 나타나고 있다[12]. 또한 2011년부터 2016년 사이의 통계청 자료를 근거로 한 연구에 의하면 지역적으로는 도시보다 농촌의 청소년에게서 자살률이 높게 나타나[10] 사회적으로 주류에 속하지 않는 여성, 농촌 거주, 후기 청소년기가 자살 생각의 고위험군임을 알 수 있다.

기존의 자살 연구는 대부분 전 연령대를 대상으로 하며, 도시와 농촌을 구분하지 않고, 성별을 통합하여 연구하고 있다. 하지만 청소년 자살 생각 연구는 다차원적으로 이루어질 필요가 있으며 성별, 연령에 따른 구분과 함께 영향요인에 대해 보다 세부적으로 탐색해야 한다[5]. 따라서 소외된 지역에 해당하는 농촌에 거주하는 후기 청소년 여성을 대상으로 자살 생각에 영향을 미치는 요인을 구체적인 초점 집단으로 하여 연구하고자 한다. 본 연구의 결과는 농촌에 거주하는 후기 청소년 여성을 대상으로 자살 생각의 영향요인에 초점을 맞춘 예방 교육을 중재하는 데 기초자료로 활용될 수 있을 것이다.

### 연구 목적

본 연구는 한국 농촌에 거주하는 후기 청소년 여성의 자살 생각에 영향을 미치는 요인을 조사하기 위한 것으로서 구체적인 목표는 다음과 같다.

1) 농촌 후기 청소년 여성의 우울, 주관적 안녕, 자살 생각의 정도를 파악한다.

2) 농촌 후기 청소년 여성의 일반적 특성에 따른 자살 생각의 차이를 확인한다.

3) 농촌 후기 청소년 여성의 우울, 주관적 안녕, 자살 생각 간의 상관관계를 확인한다.

4) 농촌 후기 청소년 여성의 자살 생각에 미치는 영향요인을 파악한다.

## Methods

Ethics statement: This study was approved by the Institutional Review Board (IRB) of Kongju National University (KNU-IRB-2019-84). Informed consent was obtained from the subjects.

### 연구 설계

본 연구는 자가보고식 설문지 조사를 기반으로 한 연구로, 농촌에 거주하는 후기 청소년 여성의 자살 생각에 영향을 미치는 영향요인을 분석하기 위한 상관성 조사연구 설계이다.

### 연구 대상

본 연구는 농촌에 거주하는 후기 청소년 여성 197명을 대상으로 하였다. 대상자의 선정 기준은 첫째, 후기 청소년기에 해당하는 만 18–22세 사이의 여성으로[5], 둘째, 농촌 지역에 거주하며, 셋째, 한국어를 이해하며 연구 목적에 동의한 자이다. 대상자의 제외 기준은 설문지의 현재 건강 문제와 과거 건강 문제의 자가 보고에 정신질환을 가지고 있다고 응답하였거나, 도시에 거주하며 학업으로 인해 일시적으로 농촌 지역에 이주해 온 자로 하였다.

G*power 3.1.9.4 프로그램을 사용하여 유의수준(α)=0.05, 독립변인 7개(우울, 주관적 안녕, 주변 사람의 자살, 구체적 자살 동기, 연령, 소득, 폭력 경험), 효과 크기 0.15, 검정력 0.80 기준으로 회귀분석 표본 크기를 산출하였을 때 103명이었다. 효과 크기는 자살 생각 관련 메타 분석의 Hedge’s g값을 참조하였다[13]. 본 연구에서는 청소년 자살관련 선행연구의 응답률인 40%와 68%의 응답률 평균을 사용하여[14] 200명의 대상자에게 설문지를 배부하고 197명(회수율 98.5%)의 자료를 분석하였다.

### 연구 도구

#### 우울

우울은 미국정신보건원이 일반인의 우울을 측정하기 위해 개발한 Center for Epidemiological Studies-Depression Scale (CES-D)를 [15] Shin 등[16]이 번역한 도구를 사용했으며, 한국어판 연구자의 승인을 받아 측정하였다. 지난 1주일 동안 1회 이하 경험한 경우인 ‘극히 드물다=0점’ 에서 지난 일주일 동안 5일 이상 경험한 경우인 ‘대부분 그랬다=3점’까지의 4점 Likert 척도로 구성되었다. 총 20문항의 가능한 점수 범위는 0–60점이며, 점수가 높을수록 우울 정도가 높은 것을 의미하고 한국인 대상 절사점으로 24점 이상은 위험군을 의미한다[16]. 도구 개발 당시의 Cronbach’s α는 .89였으며, 본 연구에서의 Cronbach’s α는 .91로 나타났다.

#### 주관적 안녕

주관적 안녕은 Suh와 Koo [11]가 개발한 Concise Measure of Subjective Well-Being (COMOSWB)으로 개발자의 승인을 받아 측정하였다. COMOSWB의 9문항 중 문항 1–3은 만족감, 문항 4–6은 긍정 정서, 문항 7–9는 부정 정서의 하위 범주로 나누어져 있다. ‘전혀 그렇지 않다=1점’에서 ‘매우 그렇다=7점’의 7점 Likert 척도를 사용하여 가능한 점수의 범위는 9–63점이며, 점수가 높을수록 주관적 안녕이 높은 것을 의미한다. 도구 개발 당시의 Cronbach’s α는 .79였으며, 본 연구에서의 Cronbach’s α는 .83으로 나타났다.

#### 자살 생각

자살 생각은 Beck 등[17]에 의해 제안된 Scale for Suicidal Ideation을 Shin 등[18]이 번역한 도구를 이용하여 한국어판 연구자의 승인을 받아 측정하였다. 도구의 구성은 총 19문항으로 ‘죽고 싶다는 생각’, ‘자살에 대한 생각’, ‘누군가에게 자살하고 싶다고 말한 경험’, ‘인생이 자살로 끝날 것이라는 생각’, ‘자살 시도 경험’ 등으로 구성되어 있으며 심각성에 따라 0점에서 3점 사이의 척도로 구성되었다. 점수의 범위는 0–57점으로, 점수가 높을수록 자살 생각에 대한 경험이 많은 것을 의미한다. 도구 개발 당시의 Cronbach’s α는 .83이었으며, 본 연구에서의 Cronbach’s α는 .87로 나타났다.

#### 자살 관련 특성

자살 관련 특성은 Renberg와 Jacobsson [19]에 의해 개발되고 Park과 Kim [20]에 의해 타당화된 한국어판 자살태도 도구 중 연구자의 승인을 받고 구체적 자살 계획 1문항과 주변 사람의 자살 경험 1문항, 총 2 문항을 사용하였다. 구체적 자살 계획은 지난 1년간 자살을 구체적으로 계획한 적이 있는지에 대한 질문으로, 0점 ‘매우 그렇지 않다’에서 4점 ‘매우 그렇다’로 답변하도록 하였다. 주변 사람의 자살 경험은 가까운 사람 중에 자살한 사람이 있는지에 대한 질문으로 1점 ‘전혀 없다’, 2점 ‘자살을 생각하는 사람이 있다’, 3점 ‘자살을 시도한 사람이 있다’, 4점 ‘자살한 사람이 있다’, 5점 ‘자살하려다 실패한 사람이 있다’, 6점 ‘자살을 시도한 사람과 자살한 사람이 모두 있다’였다.

### 자료 수집

자료 수집은 2020년 2월에 경기도 안성시, 충청남도 공주시, 전라북도 전주시에 거주하는 여성 청소년을 대상으로 수행하였다. 연구자 2인에 의해 2개의 고등학교, 2개의 대학교, 1개의 교회, 1개의 지역사회 복지관에서 200명에게 임의 표집 방법으로 설문지를 조사하였다. 지역 선정은 Kim [21]의 여성 청소년 자살률 지역분포도에서 상위 1–2단계에 포함된 농촌 지역을 선정하였다. 고등학교에서 자료를 수집하는 경우에는 학교장과 담임 교사로부터, 대학교에서 자료를 수집하는 경우는 학과장과 담당 교수로부터 허가를 받아 연구자가 자료수집을 진행하였다. 설문지는 밀봉이 가능한 봉투에 넣어 전달하고 수거 시 밀봉하여 제출하도록 하였다. 설문지 조사 장소는 교실, 강의실, 교회 회합실, 복지관 로비였고, 설문 소요시간은 5–10분이었다. 참여자에게는 설문 작성 중에라도 철회가 가능하며, 설문지는 연구 목적 외에는 사용되지 않음과 연구가 끝나고 폐기됨을 설명하였다. 참여자에게는 설문지 수거 시 자살 상담 핫라인 번호 스티커를 배부하고, 도움 요청 방법을 알려주었다. 또한 정서적 지지와 상담을 받을 수 있도록 연결해줄 수 있음을 설명하고 원하는 경우 연락하도록 설명하였다.

### 자료 분석

자료는 SPSS Statistics Win version 24.0 program (IBM Corp., Armonk, NY, USA)을 이용하여 다음의 통계 방법으로 분석하였다.

1) 우울, 주관적 안녕, 자살 생각의 정도는 빈도, 백분율, 평균, 표준편차를 구하였다.

2) 일반적 특성에 따른 자살 생각의 차이는 t-test와 ANOVA를 시행하였다.

3) 우울, 주관적 안녕, 자살 생각 간의 상관관계는 Pearson correlation coefficient를 구하였다.

4) 자살 생각에 미치는 영향요인 분석은 변수의 정규성 분포를 Kolmogorov-Smirnov 검정으로 확인하고, 변수 간의 다중 공선성 검정을 위해 분산팽창지수(variance inflation factor, VIF)와 Durbin-Watson test를 시행한 후 입력 방식의 위계적 회귀분석을 시행하였다.

## Results

### 우울, 주관적 안녕, 자살 생각의 정도와 일반적 특성에 따른 자살 생각의 차이

대상자 총 197명 중 20대는 57.9% (n=114)였으며, 소득 수준은 ‘상’이 42.1% (n=83)였다. 폭력 노출 경험이 있다고 응답한 경우가 24.9% (n=49)였다. 가정 폭력을 경험하였다고 응답한 경우는 10.7% (n=21)였다. 주변 사람의 자살 경험 평균±표준 편차는 4.46±2.19점, 구체적 자살 계획 점수는 0.53±0.78점이었다(Table 1). 우울은 평균 15.88±10.13점으로 중등도 수준이었으며, 24점 이상의 우울군은 44명(22.3%)으로 우울군의 우울점수 평균은 31.41±5.47, 비우울군의 평균은 11.48±5.89였다. 주관적 안녕은 총점 평균이 38.27±9.25점, 하위 영역인 만족감, 긍정적 정서, 부정적 정서의 평균은 각각 14.42±3.76점, 14.09±3.90점, 11.24±3.98점이었다. 자살 생각은 평균 24.24±5.27점으로 중등도 수준이었으며, 24점 이상의 위험군은 32.7%였다(Table 2).

연령이 10대인 경우 20대에 비해 자살 생각 점수가 더 높았다(t=2.65, *p*=.009). 소득에 따른 자살 생각의 차이는 통계적으로 유의하지 않았다. 폭력 노출 경험이 있는 대상자는 없는 대상자에 비해 자살 생각 점수가 더 높았다(t=–2.37, *p*=.018). 가정 폭력 노출 경험에 따른 자살 생각의 차이는 통계적으로 유의하지 않았다(Table 1).

### 우울, 주관적 안녕, 자살 생각 간의 상관관계

자살 생각은 연령(r=–.21, *p*=.003), 폭력 경험(r=.15, *p*=.029), 구체적 자살 계획(r=.65, *p*<.001), 우울(r=.39, *p*<.001), 주관적 안녕(r=–.46, *p*<.001)과 관련성이 있었다. 구체적 자살 계획은 주변 사람의 자살 경험(r=.30, *p*<.001), 우울(r=.35, *p*<.001)과 관련성이 있었다. 주관적 안녕은 주변 사람의 자살 경험(r=–.20, *p*=.005), 구체적 자살 계획(r=–.37, *p*<.001), 우울(r=–.58, *p*<.001)과 관련성이 있었다(Table 3).

### 자살 생각에 미치는 영향요인

Kolmogorov-Smirnov 검정 결과 z=.144였고, 독립변수와 종속변수의 정규 P-P 도표 기울기가 45도로 나타나 정규성과 선형성이 확인되었다. 공차 한계는 모두 0.1 이상의 수치를 보였고, 모형의 VIF 검정 결과 1.101–1.590으로 다중 공선성이 없었으며, Durbin-Watson test 결과 1.992–2.007로 잔차의 독립성이 있었고, 산점도가 0을 중심으로 고르게 퍼져 있어 등분산성이 확인되어 모형은 회귀분석에 적합하였다. 소득 수준은 ‘낮음’을 1로 dummy 처리하여 회귀분석에 투입하였다. 입력 방식의 위계적 회귀분석 결과 모형 1에서 독립변수 3개가 입력되었고 모형은 유의하였으며(F=21.63, *p*<.001), 우울(β=.20, *p*=.010), 주관적 안녕(β=–.34, *p*<.001), 소득 수준(β=–1.27, *p*=.046)이 자살 생각에 영향을 미쳤고 설명력은 23%였다(F=21.63, *p*<.001). 모형 2에서는 구체적 자살 계획이 입력되었고 모형은 유의하였으며(F=45.09, *p*<.001) 우울과 소득 수준은 영향력의 통계적 유의도가 사라졌다. 모형 2에서 주관적 안녕(β=–.22, *p*=.001), 구체적 자살 계획(β=.53, *p*<.001)이 자살 생각에 영향을 미쳤으며 설명력은 46%로 23%가 증가하였다(F=45.09, *p*<.001). 모형 3에서는 주변 사람의 자살 경험이 입력되었고 우울과 소득 수준은 지속적으로 통계적 유의도가 없었다. 모형 3에서 주관적 안녕(β=–.23, *p*=.001), 구체적 자살 계획(β=.56, *p*<.001), 주변 사람의 자살 경험(β=.12, *p*=.035)이 자살 생각에 영향을 미쳤으며 설명력은 48%로 2%가 증가하였다(F=37.62, *p*<.001) (Table 4).

## Discussion

본 연구는 농촌에 거주하는 후기 청소년 여성의 자살 생각에 영향을 미치는 요인을 탐색함으로써 여성 청소년의 자살 예방에 도움이 되는 기초 자료를 제공하고자 수행되었다. 본 연구에서 밝혀진 후기 청소년 여성의 자살 생각에 대한 영향요인은 구체적 자살 계획, 주관적 안녕, 주변 사람의 자살 경험의 순으로 나타났다. 본 장에서는 여성 청소년의 자살 생각의 관련요인과 영향요인을 중심으로 논하고자 한다.

본 연구의 결과 자살 생각의 정도는 총점 평균 24.24점으로 동일 도구로 측정한 국외 여성 청소년의 16.01점에 비해 높았으며[8], 국내 여성 청소년인 10.05점에 비해서도 높았다[6]. 24점 이상의 점수는 자살 생각을 매우 많이 하고 있음을 나타낸다는 보고를 고려하면[6] 자살률이 높은 농촌 여성 청소년의 자살 생각이 심각함을 알 수 있다. 연령대별로는 20대에 비해 10대 연령에서 자살 생각의 정도가 더 높았다. 이는 선행 연구들과 일관된 결과이며, 그 원인을 학업 스트레스로 많이 꼽고 있다. 2019년 청소년 통계에 따르면 13–18세의 경우 학업 스트레스, 19–24세는 직장 스트레스가 가장 높았으며, 여성의 스트레스는 남성에 비해 12.2% 더 높았다[22]. 그러므로 본 연구에서 분석하지 못한 자살 생각의 영향요인으로 학업과 직업 스트레스를 추후에 연구해 볼 필요가 있다.

본 연구에서 폭력 경험과 가정 폭력의 경험 여부에 따른 자살 생각 정도의 차이를 살펴본 결과, 폭력 노출 경험이 있는 여성 청소년은 그렇지 않은 경우에 비해 자살 생각 점수가 더 높은 것으로 나타나, 학교 따돌림 등의 폭력 경험을 가진 피학대 청소년의 자살 생각이 높은 수준이라는 선행 연구와 일치하였다[9]. 하지만 가정 폭력의 노출 경험 유무에 따라 자살 생각의 차이가 통계적으로 유의하게 나타나지 않았다. 이는 가정 폭력 경험을 가진 청소년이 그렇지 않은 청소년에 비해 자살 생각이 높았다는 연구 결과와는 다르지만[21], 학교 폭력에 비해 가정 폭력의 통계적 유의성이 낮다고 하는 보고와 일맥상통한다[23].

경제적 요인을 살펴보면 차이 검증에서 소득 수준 간에 자살 생각의 차이가 없어 기존 연구에서는 소득이 낮을수록 자살 생각이 높았다는 연구 결과가 보고와[24] 차이가 있었다. 한편, 청소년 자살 잠재 위험군에서는 가정 경제 스트레스가 영향요인이었으나, 자살 고위험군에서는 영향을 미치지 않았다는 보고도 있다[25]. 본 연구에서도 낮은 소득 수준이 회귀모형 1에서 자살 생각에 영향을 주었고, 2, 3모형에서는 영향력이 사라졌다. 가정의 소득 수준이 우울, 주관적 안녕 등의 타 요인에 비해서 높은 영향력을 보이지 않았다. 또한 가정의 소득 수준과 우울, 주관적 안녕감, 구체적 자살 계획, 주변 사람의 자살 경험, 자살 생각과도 유의한 상관관계를 보이지 않았다. 본 연구에서는 여성 청소년에게 가정의 소득 수준은 자살 생각에 미치는 영향이 타 요인에 비해 비중이 적게 나타났지만, 한 문항으로만 측정했기 때문일 수 있어 추후 연구를 요한다.

우울은 자살 생각의 가장 강력한 영향변수로 연구되어 왔다[23]. 본 연구에서는 회귀모형 1에서 유의한 영향변수로 나타났으며, 상관관계 분석에서는 우울이 구체적 자살 계획, 자살 생각과 유의한 양의 상관관계를, 주관적 안녕감과는 유의한 음의 상관관계를 나타내었다. 우울은 스트레스 사건 이후 오랜 시간 동안 진행되고 있으며, 본인이 우울하다는 상태를 인지하지 못한 경우도 있고, 우울을 중재할 수 있는 초기의 상태를 지나 심각한 상태에 이르는 경우가 많다[26]. 그러므로 농촌에 거주하는 여성 청소년의 우울이 만성화되기 전에 초기 단계에 사정하여 잠재적 위험이 있는 대상자의 심리적 중재가 필요할 것이다.

본 연구에서 회귀분석 결과 모형에 투입되었을 때 23%의 설명력 증가를 가져와 자살 생각에 가장 영향을 크게 주는 요인이 구체적 자살 계획으로 나타났다. 청소년의 자살률은 남성이 여성에 비해 높지만, 자살 시도 비율과 자살 생각 정도는 여성이 남성에 비해 높았다[4]. 여성 청소년은 자살 생각의 내재화 정도가 높고 남성 청소년에 비해 자살 생각의 감소 궤적이 적어 자살 생각이 쉽게 사라지지 않는 특징을 가지고 있다[27]. 청소년의 자살 시도 대다수가 사전에 계획된 것이 아니라 충동적이라는 과거의 연구 결과가 있었으나, 본 연구에서는 구체적인 자살 계획이 가장 큰 요인으로 나타나 청소년의 자살이 현실도피적, 충동적, 일시적인 스트레스에 의한 반응이라는 연구 결과들과는 상반된 결과를 보여주고 있다[28]. 이는 청소년의 자살 예방책을 마련하는 데 중요한 단서가 될 수 있는 것으로, 청소년의 자살이 충동적이라는 관점보다는 예측 가능하다는 데에 무게를 두고 적극적으로 중재해야 할 것이다. 본 연구의 결과는 청소년 자살 예방을 위한 정책 및 시스템 구축에 있어, 청소년들에게 구체적 자살 계획이 있는지를 선별하고 조기에 예방적 개입과 처방이 가능하며, 적절한 도움을 줄 수 있다는 점을 시사한다.

본 연구에서 두 번째로 영향력이 높았던 독립변수는 주관적 안녕이었는데, 주관적 안녕은 높은 긍정적 정서와 만족감, 낮은 부정적 정서로 이루어져 있다. 본 연구의 상관관계 분석에서도 주관적 안녕은 주변 사람의 자살 경험, 구체적 자살 계획과 약한 상관관계를, 우울과는 중등도의 상관관계를 가지고 있었다. 이 결과는 주관적 안녕감이 대상자의 자살 생각에 보호요인으로 작용할 수 있다는 시사점을 준다. 선행연구[29]에서 청소년의 자살 생각 보호요인으로는 낙관성, 자아 존중감, 정서 조절이 있었던 것과 일치하나, 위험요인으로 보고된 학업 스트레스나 자살 고위험군에게 위험요인이 한꺼번에 닥칠 경우 자살 위험이 높아진 집중적 특성은 측정하지 못해 추후 연구를 요한다.

본 연구에서 주변 사람의 자살 경험은 자살 생각에 세 번째로 영향력이 높은 변수였다. 주변 사람의 자살 경험 정도는 6점 척도에서 평균 4.46점으로 매우 높은 수준이었다. 국내 자살자에 대한 심리 부검에서 자살자의 45.5%가 부모 혹은 조부모의 자살 시도를 경험한 것으로 나타났고, 친척 중 자살 사망 경험이 36.4%로 나타났다[2]. 영국 연구에서는 가까운 이의 자살을 경험한 대상자의 49%가 자살 생각을 하였으며, 9%가 자살 시도를 하였다[14]. 사회적으로 베르테르 효과(the Werther effect)라고 불리는 유명인의 자살 사건은 민감한 청소년기에 모방 자살의 위험을 증가시킬 수 있다. 가족이나 친구, 친척의 자살을 경험한 사람은 자살 생존자로 분류되고 있다[30].

연구 결과 농촌에 거주하는 후기 청소년 여성의 자살 생각에 대한 영향요인은 구체적 자살 계획, 주관적 안녕감, 주변 사람의 자살 경험 정도의 순이었다. 20대보다는 10대, 폭력의 경험이 없는 경우보다는 있는 경우에 자살 생각이 더 높았으며, 우울감이 높을수록 자살 생각이 많았다. 결론적으로 농촌에 거주하는 여성 청소년의 자살 예방을 위해서 잠재적인 자살 위험군을 선별하려면 구체적 자살 계획, 자살 생각 및 우울, 폭력 경험을 사정할 필요가 있다. 선별된 위험군에는 주관적 안녕감의 속성인 만족감, 긍정적 정서를 높이고 부정적 정서를 줄이는 교육과 세밀한 정서적 중재를 통하여, 농촌에 거주하는 여성 청소년의 자살 생각을 감소시키는 노력을 기울여야 할 것이다. 친구, 친척, 가족의 자살 사건 경험이 있는 여성 청소년은 자살 생존자에 해당하므로 그에 준하는 자살 예방 계획과 중재를 실시할 필요가 있으며, 자살 생존자에 대한 사적인 비밀 유지 및 인권 보호적 상담에 관하여 철저히 보호하는 장치를 마련해야 할 것이다. 또한 자살 생존자 예방적 중재에 농촌에 거주하는 여성 청소년을 우선적으로 배려해야 할 것이며, 미디어의 자살 사건 보도는 청소년에 대한 영향을 고려하여 신중을 기해야 할 것이다.

본 연구의 의의는 농촌에 거주하는 후기 청소년 여성의 자살 예방 중재에 활용할 수 있는 영향요인을 파악하였다는 데 있다. 2019년 청소년 통계에 의하면 청소년의 상담 대상은 주로 친구(49.1%)이고, 그 다음이 부모(28.0%), 스스로 해결(13.8%)인 것으로 나타났으며[4], 낙심하거나 우울할 때 도움을 받을 수 있는 사람이 전혀 없다고 응답한 청소년이 10.7%나 되었다[4]. 청소년의 고민 상담에 스승은 0.9%, 전문 상담자를 포함한 기타가 0.8%로 나타나 전문적 중재가 부족한 현실이다[4]. 청소년에 대한 자살 예방 중재는 지원 서비스와 동시에 진행되어야 하며, 죄책감과 수치심을 고려하여[29] 본 연구에서 도출된 결과를 토대로 세심하게 이루어져야 한다.

본 연구의 제한점은 첫째, 자살률이 높은 3개 지역의 농촌에서 표집한 결과로 전국의 농촌 지역을 대표하지 않는다는 점과, 일부의 여성 청소년을 대상으로 편의추출하여 일반화할 수 없다는 점이다. 둘째, 설문지에 기초한 조사 연구이므로 대상자의 응답에 의존하여 민감한 질문에 대한 응답의 오류가 존재할 수 있다. 셋째, 폭력 경험, 구체적 자살 계획, 주변인의 자살 경험 문항이 각각 하나의 문항으로 이루어져 보다 타당화된 도구로 측정할 필요가 있다.

본 연구의 제언은 다음과 같다. 첫째, 여성 청소년의 학업 스트레스와 직업 스트레스를 측정하여 자살 생각의 영향요인을 분석하는 연구를 제언한다. 둘째, 자살의 위험요인과 함께 보호요인을 조사하여, 예방 중재에 도움이 되는 매개변수를 파악하는 연구를 제언한다. 셋째, 본 연구에서 밝혀진 영향요인을 참고하여 농촌에 거주하는 후기 청소년 여성의 특성에 세밀하게 적합한 중재를 개발하여 적용할 것을 제언한다.

## Figures and Tables

**Table 1.** Suicide ideation according to participants’ characteristics (N=197)

Variable	Categories	n (%)	Mean±SD	F/t	*p*
Age (year)	18–19	83 (42.1)	25.43±5.22	2.65	.009
	20–22	114 (57.9)	23.44±5.19		
Household income	High	60 (30.5)	24.91±5.86	1.34	.265
	Medium	65 (33.0)	24.37±5.13		
	Low	72 (36.5)	23.42±4.65		
Ever experienced violence	Yes	49 (24.9)	25.82±5.63	–2.37	.018
	No	148 (75.1)	23.77±5.08		
Ever experienced domestic violence	Yes	21 (10.7)	26.19±5.08	–1.76	.079
	No	176 (89.3)	24.05±5.20		
Suicide of a close person			4.46±2.19		
Suicide plan			0.53±0.78		

**Table 2.** Suicide ideation according to depression and subjective well-being (N=197)

Variable	Categories	n (%)	Mean±SD	Min-Max
Depression	Total	197 (100)	15.88±10.13	1–48
	Non-depressive (<24)	153 (77.7)	11.48±5.89	1–23
	Depressive (≥24)	44 (22.3)	31.41±5.47	24-48
Subjective well-being	Total		38.27±9.25	11–60
	Satisfaction		14.42±3.76	4–21
	Positive affect		14.09±3.90	3–21
	Negative affect		11.24±3.98	3–21
Suicide ideation			24.24±5.27	16–42

**Table 3.** Correlations among participant characteristics and suicidal ideation (N=197)

Variable	r (*p*)						
	Age	Household income^†^	Ever experienced violence^†^	Suicide of a close person	Having a specific suicide plan	Depression	Subjective well-being
Household income	.21 (.002)						
Exposure to violence	.09 (.171)	.22 (.002)					
Suicide of a close person	–.18 (.011)	–.07 (.306)	–.03(.637)				
Having a specific suicide plan	–.13 (.056)	.05 (.438)	.11 (.108)	.30 (<.001)			
Depression	–.01 (.929)	–.08 (.594)	.17 (.016)	–.13 (.051)	.35 (<.001)		
Subjective well-being	.01 (.968)	–.03 (.651)	–.17 (.013)	–.20 (.005)	–.37 (<.001)	–.58 (<.001)	
Suicidal ideation	–.21 (.003)	–.09 (.200)	.15 (.029)	.11 (.110)	.65 (<.001)	.39 (<.001)	–.46 (<.001)

† Dummy references were household income (low) and ever experienced violence (yes).

**Table 4.** Factors influencing suicidal ideation (N=197)

Variable
	Analysis Model 1			Analysis Model 2			Analysis Model 3		
	β	t	*p*	β	t	*p*	β	t	*p*
(Constant)		14.42	<.001		14.41	<.001		13.51	<.001
Depression	.20	2.59	.010	.08	1.36	.175	.08	1.34	.180
Subjective well-being	–.34	–4.47	<.001	–.22	–3.28	.001	–.23	–3.49	.001
Household income^†^	–1.27	–2.01	.046	–.07	–1.32	.190	–.07	–1.35	.179
Having a specific suicide plan				.53	9.22	<.001	.56	9.54	<.001
Suicide of a close person							.12	2.12	.035
F(*df*), *p*	21.62 (3), <.001			45.09 (4), <.001			37.62 (5), <.001		
Adjusted R^2^	.23			.46			.48		
R^2^ change (F, *p*)				.23 (44.10, <.001)			.02 (36.83, <.001)		

† The dummy variable reference for household income was (low).
